# Chinese Herbal Medicine for Psoriasis: Evidence From 11 High-Quality Randomized Controlled Trials

**DOI:** 10.3389/fphar.2020.599433

**Published:** 2021-01-22

**Authors:** Yue Luo, Jiale Chen, Le Kuai, Ying Zhang, Xiaojie Ding, Ying Luo, Yi Ru, Meng Xing, Hongjin Li, Xiaoying Sun, Bin Li, Xin Li

**Affiliations:** ^1^ Department of Dermatology, Yueyang Hospital of Integrated Traditional Chinese and Western Medicine, Shanghai University of Traditional Chinese Medicine, Shanghai, China; ^2^ Institute of Dermatology, Shanghai Academy of Traditional Chinese Medicine, Shanghai, China; ^3^ Shanghai Dermatology Hospital, Tongji University, Shanghai, China

**Keywords:** Chinese herbal medicine, psoriasis, high-quality, randomized controlled trials, meta-analysis

## Abstract

**Background:** Chinese herbal medicine (CHM) provides a theoretical basis for the treatment of psoriasis with considerable benefits and a low toxicity. The purpose of this quantitative study was to show high-quality evidence of the efficacy and safety of CHM for the treatment of psoriasis to promote its clinical application.

**Methods:** Several databases were systematically searched including PubMed, Embase, Cochrane Central Register of Controlled Trials, China Network Knowledge Infrastructure, Chinese Scientific Journals Database, and Wan Fang Database. High-quality randomized controlled trials that compared CHM with non-CHM interventions were included. The RevMan5.3 software was used to calculate risk ratios (RR) at 95% confidence intervals (CI) and conduct the meta-analysis.

**Results:** Altogether, 1,215 patients participated in this study, including 711 in the experimental group and 504 in the control group. The psoriasis area severity index (PASI) score of the CHM group was significantly lower than that of the placebo group (MD, −4.02; 95% CI, −6.71 to −1.34; *p* = 0.003). To achieve PASI-60 and PASI-75, the arrival rate of the CHM group was higher than that of the placebo group (PASI-60: RR, 3.52; 95% CI, 1.17 to 10.61; *p* = 0.03; PASI-75: RR, 9.87; 95% CI, 3.11 to 31.31; *p* = 0.0001). Furthermore, the efficacy rate was higher in patients receiving CHM than in those receiving placebo (RR, 1.72; 95% CI, 1.01 to 2.93; *p* = 0.04). The results suggested a greater impact of CHM in improving the dermatology life quality index (DLQI) of patients (MD, −2.12; 95% CI, −3.75 to −0.49; *p* = 0.01). Regarding pruritus severity, there was no significant difference between the two groups (MD, −1.90; 95% CI, −3.79 to −0.01; *p* = 0.05). The meta-analysis revealed that the recurrence rate (RR, 0.74; 95% CI, 0.32 to 1.71; *p* = 0.48) and proportion of adverse events (RR, 1.36; 95% CI, 0.95 to 1.93; *p* = 0.09) associated with using CHM were similar to those associated with using a placebo.

**Conclusion:** CHM appears safe and effective in the treatment of psoriasis and has a great positive impact on the DQLI of patients; however, CHM could not completely eliminate skin lesions, improve pruritus severity, and reduce the recurrence rate.

## Introduction

Psoriasis is an autoimmune disease characterized by excessive proliferation and abnormal epidermal differentiation at typical body sites, with the plaque type being the most common presentation. It is a chronic, recurrent, inflammatory skin disorder that presents with erythema, papules, and scales, which may be painful and itchy (Griffiths and Barker, 2007; Ramanunny et al., 2020). Approximately 0.51–11.43% of adults and 0–1.37% of children worldwide suffer from psoriasis (Michalek et al., 2017). It is not only a skin disease but has an impact on the patients’ physical and psychological quality of life. Evidence shows an association of psoriasis with arthritis, inflammatory bowel disease (Oliveira et al., 2015), metabolic syndrome (Armstrong et al., 2013), cerebrovascular diseases (Tangtatco and Lara-Carrales, 2017), and mood disorders such as depression and anxiety (Kurd et al., 2010), all of which can be considered comorbidities of psoriasis.

The etiology and pathogenesis of psoriasis has not yet been fully elucidated. It may be related to genetic and environmental factors, and an abnormal immune response. Studies have shown that psoriasis is a true T cell-mediated disease characterized by infiltration of inflammatory cells and excessive proliferation and differentiation of epidermal keratinocytes (KC) (Lowes et al., 2007). In the pathogenesis of psoriasis, KC damage in the epidermis triggers local inflammation, enhances the chemotaxis of T cells and neutrophils, and leads to KC activation and vascular endothelial dysfunction. This stimulates the production of circulating inflammatory cytokines such as tumor necrosis factor-α (TNF-α), interleukin-17 (IL-17), interleukin-6 (IL-6), interleukin-1β (IL-1β), interferon-γ (IFN-γ) and vascular endothelial growth factor (VEGF). As a result, mature psoriatic plaques develop by inducing epidermal hyperplasia, epidermal cell proliferation and leukocyte subset recruitment into the skin (Hawkes et al., 2017).

The choice of treatment for psoriasis depends on many factors including the degree of the disease, its impact on the patient's life, and the patient's perception of the disease. At present, the pharmacological treatment includes topical therapy such as emollients, vitamin D_3_ derivatives, retinoids, glucocorticoids, and oral therapy such as tretinoins, and immunosuppressants. Additionally, biological therapies are effective for patients with moderate-to-severe psoriasis (Ramanunny et al., 2020). However, most of the therapies used in psoriasis have side effects and are not appropriate for long-term use. Therefore, it is essential that psoriasis treatments remain effective with reduced side effects.

Chinese herbal medicine (CHM) provides a theoretical basis for the treatment of skin diseases and has considerable benefits and a low toxicity (Chen et al., 2018; Ru et al., 2019; Kuai et al., 2020; Li S. et al., 2020; Ru et al., 2020; Yan et al., 2020). The forms of traditional Chinese medicine include topical (cream, oil, emulsion, and ointment) and oral (powder, tablet, capsule, and soup) treatments (Dermatology Branch of Chinese Association of traditional Chinese Medicine, 2013). The syndromes of psoriasis include: 1) blood-heat syndrome, seen in the progressive stage dominated by an inflammatory reaction; 2) blood stasis syndrome, seen in the quiescent stage dominated by hyperplasia of keratinocytes; 3) blood dryness syndrome, seen in the retrogressive stage characterized by skin barrier dysfunction; 4) blazing heat toxin syndrome, seen in the erythroderma or generalized pustule type; 5) damp-heat accumulation syndrome, seen in the localized pustular type; and 6) rheumatic obstruction syndrome, seen in the arthropathy type. All psoriasis syndromes can be transformed and show mixed characteristics between them. In terms of the therapeutic effect, CHM treatment can eliminate skin lesions by removing heat and cooling the blood, nourishing and activating the blood circulation and removing blood stasis, purging fire and providing a detoxification effect, clearing heat and dispelling dampness, dispelling wind and dredge collaterals (Psoriasis Professional Committee of Dermatology and venereology Branch of Chinese Medical Association, 2019).

Evidence from clinical studies suggests that CHMs could reduce the psoriasis area severity index (PASI) score and improve the dermatology life quality index (DLQI) of patients with psoriasis (Bahraini et al., 2018; Zheng et al., 2019). Pharmacological studies demonstrated that Tanshinone IIA, the effective component of danshen (*Salvia miltiorrhiza*), can inhibit the proliferation of KC, induce apoptosis, and block the cell cycle of KC (Li F. L. et al., 2012). Drugs promoting blood circulation and removing blood stasis such as peach kernel and chuanxiong (*Ligusticum chuanxiong* Hort.) can dilate blood vessels, increase tissue blood flow, reduce blood viscosity, improve the microcirculation, and hence, promote the regression of skin lesions (Xiao et al., 2019). Additionally, several systematic reviews on the efficacy of CHM in the treatment of psoriasis have been published (Li N. et al., 2012; Deng et al., 2013; Yang et al., 2015; Zhang et al., 2015); however, most are low-quality studies. Moreover, a substantial amount of new data has been published. Therefore, the purpose of this quantitative study was to collect evidence on the efficacy and safety of CHM in the treatment of psoriasis to promote its clinical application.

## Methods

This study was conducted in accordance with the Cochrane Handbook on Systematic Review of Interventions and presented in accordance with the Preferred Reporting Items for Systematic Review and Meta-Analysis (PRISMA) Guidelines (Liberati et al., 2009) (Additional file: Supplementary Table S1). Additionally, before starting the process, the review was registered in the PROSPERO database (CRD42020204557).

### Search Strategy

We searched PubMed, Embase, Cochrane Central Register of Controlled Trials (CENTRAL), China Network Knowledge Infrastructure, China Science and Technology Journal Database, and Wan Fang Database from inception to May 31, 2020. We combined medical subject headings and free text words to retrieve all relevant studies. The following keywords were used: (“Psoriasis” OR “Psoriases” OR “Pustulosis Palmaris et Plantaris” OR “Palmoplantaris Pustulosis” OR “Pustulosis of Palms and Soles” OR “Palmoplantaris” OR “Pustulosis” OR “Pustular Psoriasis of Palms and Soles” OR “Parapsoriasis guttata”) and (“Traditional Chinese Medicine” OR “Chung I Hsueh” OR “Hsueh, Chung I” OR “Traditional Medicine, Chinese” OR “Zhong Yi Xue” OR “Chinese Traditional Medicine” OR “Chinese Medicine, Traditional” OR “Chinese drugs” OR “Chinese herbal medicine” OR “Chinese herbal drug”) (Additional file: Supplementary Table S2). Moreover, we searched the Chinese Clinical Trial Registry (http://www.chictr.org.cn/index.aspx) and Clinical Trials (http://www.clinicaltrials.gov) websites to identify protocols of high quality randomized controlled trials (RCTs).

### Inclusion and Exclusion Criteria

The inclusion criteria were as follows: 1) patients presenting specific diagnostic criteria of psoriasis regardless of age, gender, or ethnicity; 2) RCTs that compared CHM with non-CHM interventions; 3) high-quality RCTs with a Jadad score ≥4 in efficacy and safety analysis; and 4) trials must meet the criteria of double blindness. Studies were excluded if they met the following exclusion criteria: 1) participants with comorbidities; 2) different drug forms used in the experimental and control group; and 3) co-interventions that used anti-psoriatic drugs other than CHM.

### Data Extraction

Three researchers (LK, YZ, and XD) carefully screened the qualified articles according to the predetermined inclusion and exclusion criteria. Two researchers (YL and JC) completed the self-designed data extraction form which included the name of the first author, year of publication, sample size, age and gender of participants, duration, intervention of experimental and control group, course of treatment, adverse events, and outcomes.

### Outcome Measures

The primary outcome was an improvement of the PASI score after treatment, which is a quantitative rating score that measures the severity of psoriatic lesions based on area coverage and plaque appearance including scaling, infiltration, and erythema. The secondary outcomes included the efficiency, DLQI, visual analog scale (VAS) scores for the intensity of itching, recurrence rate, and adverse events (AEs).

### Risk of Bias in Individual Studies

For each included study, two investigators (YL and YR) completed the Jadad scale used specifically for the quality of the evaluation method. A third-party researcher (MX) was consulted whenever there was a disagreement between the two investigators. Four dimensions of the Jadad scale (total seven points) were applied in this research, namely randomization, concealment, blind method, and reports of withdrawals and dropouts. Trials scoring 1–3 points were considered low quality and 4–7 as high quality.

### Statistical Analysis

We synthesized the results in the meta-analysis using RevMan5.3 software provided by the Cochrane Collaboration. Risk ratios (RR) with 95% confidence intervals (CI) were evaluated for dichotomous data, continuous data, mean difference (MD), and standard mean difference (SMD) were used. Across the trials, a fixed-effects model was used if there was homogeneity (*p* >0.1, I^2^<50%); otherwise, a random-effect model was applied. *p* values less than 0.05 were considered statistically significant.

## Results

### Included Studies

**FIGURE 1:**
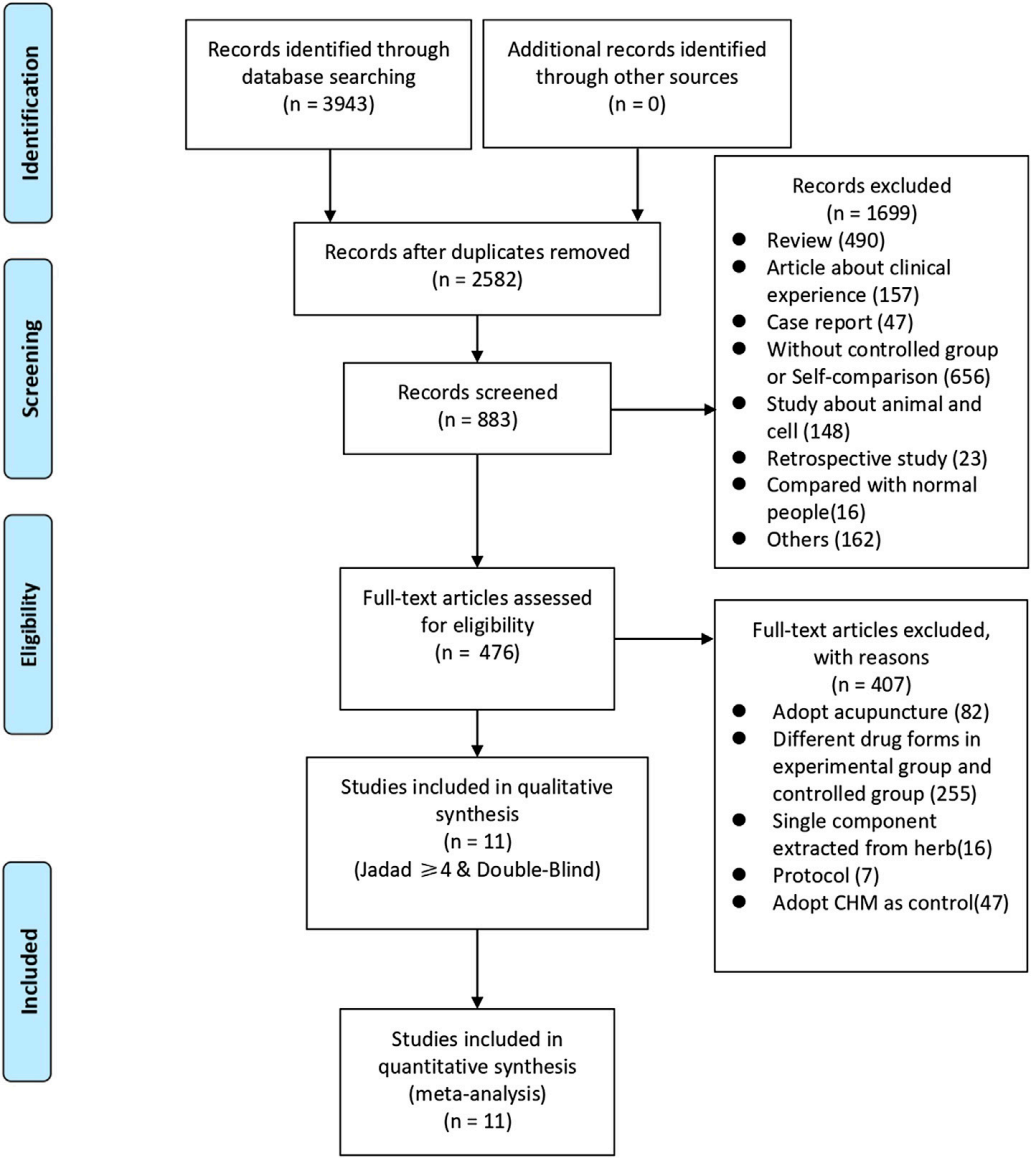
Flowchart of search strategy and study selection, according to the Preferred Reporting Items for Systematic Reviews and Meta-analyses (PRISMA) guidelines.

We identified 3,943 studies after a preliminary search of six databases; 1,361 repetitive articles were excluded, and 1,699 articles were deleted after reading the titles and abstracts. Among the remaining 883 studies, 407 were excluded since 82 adopted acupuncture in the experimental group, 255 used alternate forms of medicine, 16 adopted single-component herb extracts, seven articles were protocols of RCTs, and 47 used CHM in the control group. Finally, the remaining 476 studies were evaluated using the Jadad scores. Eleven studies with a Jadad scale ≥4 points met the inclusion criteria (Wang, 2003; Zhou, 2011; Zhou, 2012; Chen, 2016; Yao et al., 2016; Li et al., 2017; She, 2017; Lv, 2018; Zhang, 2018; Mao et al., 2019; Li B. Y. et al., 2020). Eight trials were published in Chinese and three in English. The flowchart was created to briefly illustrate the screening process (Figure 1).

### Study Characteristics

A total of 1,215 patients participated in this study, including 711 in the experimental group and 504 in the control group. All 11 trials used a placebo as control while the forms of the intervention drugs were not identical. Four trials (Zhou, 2011; Zhou, 2012; Zhang, 2018; Mao et al., 2019) used decoctions, four (Yao et al., 2016; She, 2017; Lv, 2018; Zhang, 2018) used particles, one (Li et al., 2017) used ointments, and two (Wang, 2003; Li B. Y. et al., 2020) used capsules. Meanwhile, the treatment course ranged from 4 to 12 weeks.

PASI scores were recorded as the main outcome in all 11 trials: eight reported detailed scores (Wang, 2003; Zhou, 2011; Zhou, 2012; Li et al., 2017; She, 2017; Lv, 2018; Zhang, 2018; Mao et al., 2019), 5 (Chen, 2016; Yao et al., 2016; Li et al., 2017; Lv, 2018; Zhang, 2018) reported the number of patients who achieved PASI-50, 1 (Chen, 2016) reported on PASI-60, 4 (Chen, 2016; Lv, 2018; Zhang, 2018; Li B. Y. et al., 2020) reported on PASI-75, and 2 (Zhang, 2018; Li B. Y. et al., 2020) reported on PASI-90, which signify a PASI score reduction of at least 50, 60, 75, 90%. Three trials (Zhou, 2011; Zhou, 2012; She, 2017) assessed the efficacy rate, three (Zhou, 2012; Lv, 2018; Zhang, 2018) measured DLQI scores, and three reported VAS scores. Only three articles (Yao et al., 2016; Lv, 2018; Mao et al., 2019) calculated the recurrence rates. In terms of safety, adverse events were recorded in all trials. A summary table of preparations and the species or concentration of included CHMs was presented in Table 1.

**TABLE 1:** Summary of the characteristics of the included trails.

Author Year	Sample Size		Age (years) (Mean ± SD)		Gender (M/F)		Duration of psoriasis (Mean ± SD)		Intervention		Course of treatment	Adverse events		0utcome
	E	C	E	C	E	C	E	C	E	C		E	C	
Wang (2003)	25	27	26.68 ± 12.3	28.62 ± 14.23	16/9	20/7	56.68 ± 40.23 m	61.24 ± 39.12 m	CHM Capsule	Placebo	4w	4	0	PASI, efficiency, AEs
Zhou (2011)	235	115	38.13 ± 12.47	39.50 ± 13.07	146/89	74/41	NR	NR	CHM decoction	Placebo	8W	10	2	PASI, efficiency, AEs
Zhou (2012)	35	27	34.11 ± 9.15	31.04 ± 7.88	16/19	11/16	3.97 ± 5.92 y	3.70 ± 5.78 y	CHM decoction	Placebo	8W	6	1	PASI, DLQI, VAS, efficiency, AEs
Chen (2016)	50	24	39.5 ± 12.45	36.3 ± 11.38	36/14	17/7	15.6 ± 10.83 y	15.2 ± 8.56y	CHM decoction	Placebo	8W	1	0	PASI 50, PASI 60, PASI 75, AEs
Yao et al. (2016)	8	10	45.43 ± 11.84	41.60 ± 13.24	7/0	8/2	144.57 ± 73.77 m	86.20 ± 54.02 m	CHM decoction + topical sequential treatment	Placebo + topical sequential treatment	12W	1	2	PASI50, RER, AEs
Li et al. (2017)	143	135	40 ± 13	36 ± 12	78/65	74/61	NR	NR	CHM Ointment	Placebo	4W	0	0	PASI, RER, AEs
She (2017)	81	40	30.12 ± 6.31	28.97 ± 5.03	41/40	21/19	11.87 ± 4.49 y	10.94 ± 5.38 y	CHM Particle	Placebo	12W	2	1	PASI, efficiency, AEs
Lv (2018)	38	37	42.55 ± 10.86	38.62 ± 13.65	34/4	31/6	9.68 ± 6.04 y	9.78 ± 6.31 y	CHM Particle	Placebo	12W	18	16	PASI, PASI50, PASI 75, DLQI, VAS, RER, AEs
Mao et al. (2019)	30	20	28.13 ± 9.71	31.50 ± 10.13	18/12	13/7	32.7 ± 9.3 m	36.5 ± 8.7 m	CHM decoction	Placebo	6W	0	0	PASI, AEs
Zhang (2018)	40	40	40.73 ± 10.90	43.50 ± 11.75	33/7	31/9	NR	NR	CHM Particle	Placebo	4W	11	14	PASI, PASI50, PASI75, PASI90, DLQI, VAS, AEs
Li et al. (2020a)	27	29	37.74 ± 10.50	41.45 ± 14.40	16/11	17/12	NR	NR	CHM Capsule	Placebo	4W	6	1	PASI50, PSAI75, PASI90, AEs

Author Year	TCM Syndrome (E/C)	Curative effect	Name of herbs	Compositions (Species, source, concentration)	Usage	Quality control reported? (Y/N)
Wang (2003)	NR	Clearing heat and detoxifying, removing blood stasis and removing spots, removing wind, dampness and itching	Compound Indigo Naturalis Capsules	Powder of leaves of *Strobilanthes cusia* (Nees) Kuntze, Whole plant of *Portulaca oleracea* L., Rhizome of *Smilax glabr*a Roxb, Root bark of *Dictamnus dasycarpus* Turcz., Root of *Angelica dahurica* var. formosana (H.Boissieu) Yen, Root of *Lithospermum erythrorhizon* Sieb. et Zucc., Rhizome of *Dryopteris crassirhizoma* Nakai, Whole plant of *Taraxacum mongolicum* Hand-Mazz., Root and rhizome of Salvia miltiorrhiza Bunge, Root and rhizome of *Dioscorea collettii* var. hypoglauca (Palib.) S.J.Pei & C.T.Ting, Fruit of *Prunus mume* (Siebold) Siebold & Zucc., Fruit of *Schisandra chinensis* (Turcz.) Baill., Fruit of Crataegus pinnatifida Bunge, Massa medicata fermentata	0.5 g*4 tid	Y-repared according to ChP Z20010157
Zhou (2011)	Blood heat syndrome (78/39)	Cooling blood and detoxification	Cooling Blood and detoxification decoction	Rhizome of *Smilax glabra* Roxb 30g, Flower of *Sophora japonica* L. 15g, Root of *Lithospermum erythrorhizon* Sieb. et Zucc. 10g, Rhizome of *Paris polyphylla* 9g, Root of *Rehmannia glutinosa* (Gaertn.) DC. 15g, Root bark of *Dictamnus dasycarpus* Turcz. 10g, Root of *Paeonia veitchii* Lynch 10g, [Pharmacy Department of Beijing Hospital of traditional Chinese Medicine]	100 ml bid	Y- Prepared according to ChP; Hospital preparation
	Blood dryness syndrome (79/38)	Nourishing blood and detoxification	Nourishing Blood and detoxification decoction	Root and rhizome of *Salvia miltiorrhiza Bunge* 15g, Root of *Angelica sinensis* (Oliv.) Diels. 15g, Root of *Rehmannia glutinosa* (Gaertn.) DC. 15g, Root of *Ophiopogon japonicus* (L. f.) Ker-Gawl. 10g, Root of *Scrophularia ningpoensis* Hemsl. 15g, dried lianoid stem of *Spatholobus suberectus* Dunn 15g, Rhizome of *Smilax glabra* Roxb 30g, [Pharmacy Department of Beijing Hospital of Traditional Chinese Medicine]	100 ml bid	
	Blood stasis syndrome (78/38)	Promoting blood circulation and detoxification	Promoting Blood Circulation and detoxification decoction	Whole grass of *Hedyotis diffusa* Willd. 30g, Rhizome of *Curcuma zedoaria* (Christm.) Roscoe 10g, Wing-shaped cork of *Euonymus alatus* (Thunb.) Siebold 10g, Flower of *Carthamus tinctorius* L. 10g, dried lianoid stem of *Spatholobus suberectus* Dunn 30g, Kernel of *Amygdalus persica L.* 10g, Root and rhizome of *Salvia miltiorrhiza* Bunge 15g, [Pharmacy Department of Beijing Hospital of traditional Chinese Medicine]	100 ml bid	
Zhou (2012)	Blood heat syndrome (35/27)	Cooling blood and promoting blood circulation	Liangxuehuoxue Complex Prescription	Leaf of *Isatis tinctoria L.* 15g, Root of *Rehmannia glutinosa* (Gaertn.) DC. 30g, Root of *Scutellaria baicalensis Georgi* 12g, Root of *Lithospermum erythrorhizon* Sieb. et Zucc. 9g, Root and rhizome of *Salvia miltiorrhiza* Bunge 12g, Root of *Paeonia veitchii* Lynch 6g, Root bark of *Paeonia suffruticosa* Andr. 9g, Root of *Angelica sinensis* (Oliv.) Diels. 12g, Rhizome of *Smilax glabra* Roxb 30g	100 ml bid	NR
Chen (2016)	Blood heat syndrome (15/10)	Cooling blood and detoxification	Cooling Blood and detoxification decoction	Rhizome of *Smilax glabra* Roxb, Flower of *Sophora japonica* L., Root of *Lithospermum erythrorhizon* Sieb. et Zucc., Root of *Rehmannia glutinosa* (Gaertn.) DC., Root of *Paeonia veitchii* Lynch, Root bark of *Paeonia suffruticosa* Andr., Rhizome of *Imperata cylindrica* (L.) Beauv., Root bark of *Dictamnus dasycarpus* Turcz., Root of *Saposhnikovia divaricata* (Turcz. ex Ledeb.) Schischk., Whole grass of *Hedyotis diffusa* Willd., Rhizome of *Paris polyphylla*, Root of *Isatis tinctoria L.*, [Beijing Institute of traditional Chinese Medicine]	200 ml	Y- Prepared according to ChP; Hospital preparation
	Blood dryness syndrome (17/5)	Nourishing blood and detoxification	Nourishing Blood and detoxification decoction	Root of *Angelica sinensis* (Oliv.) Diels., Root of *Rehmannia glutinosa* (Gaertn.) DC., Root and rhizome of *Salvia miltiorrhiza* Bunge, dried lianoid stem of *Spatholobus suberectus* Dunn, Root of *Ophiopogon japonicus* (L. f.) Ker-Gawl., Root of *Scrophularia ningpoensis* Hemsl., Root of Trichosanthes kirilowii Maxim., Rhizome of *Smilax glabra* Roxb, Whole grass of *Hedyotis diffusa* Willd., Rhizome of *Paris polyphylla*, Rhizome of *Atractylodes lancea* (Thunb.) DC., Fruit of *Tribulus terrestris* L., [Beijing Institute of traditional Chinese Medicine]	200 ml	
	Blood stasis syndrome (4/3)	Promoting blood circulation and detoxification	Promoting Blood Circulation and detoxification decoction	Root of *Angelica sinensis* (Oliv.) Diels., Rhizome of *Curcuma zedoaria* (Christm.) Roscoe, Root and rhizome of *Salvia miltiorrhiza* Bunge dried lianoid stem of *Spatholobus suberectus* Dunn, Kernel of *Amygdalus persica L.*, Root of *Scrophularia ningpoensis* Hemsl., Root and rhizome of *Clematis chinensis* Osbeck, Branch of *Cinnamomum cassia* (L.) J. Presl, Rhizome of *Atractylodes lancea* (Thunb.) DC., Wing-shaped cork of *Euonymus alatus* (Thunb.) Siebold, Whole grass of *Hedyotis diffusa* Willd., Rhizome of *Paris polyphylla* [Beijing Institute of traditional Chinese Medicine]	200 ml	
Yao et al. (2016)	NR	Promoting blood circulation, removing blood stasis and removing spots, removing wind, dampness and itching	PSORI-CM01	Root of *Paeonia veitchii* Lynch, Rhizome of *Curcuma zedoaria* (Christm.) Roscoe, Whole plant of *Sarcandra glabra* (Thunb.) Nakai, Root and rhizome of *Glycyrrhiza uralensis* Fisch. ex DC., Fruit of *Prunus mume* (Siebold) Siebold & Zucc., Root of *Lithospermum erythrorhizon* Sieb. et Zucc., and Rhizome of *Smilax glabra* Roxb, [Pharmaceutical department of Chinese herbal medicine of GPHCM]	100 ml bid	Y- Prepared according to ChP; Hospital preparation
Li et al. (2017)	Blood heat syndrome (143/135)	Clearing excess heat and dampness, and relieving swelling and pain.	Pulian Ointment	Powder of bark of *Phellodendron amurense* Rupr. 50g, Powder of root of *Scutellaria baicalensis* Georgi 50g and white petroleum jelly 400 g, [China-Japan Friendship Hospital]	2 mm larger than the skin lesion area bid	Y- Prepared according to ChP; Hospital preparation
She (2017)	Blood dryness syndrome (44/20)	Calming the mind and relieving itching (Blood dryness)	No.1 Calm-the-Mind-and-Relieve-Itching Formula	Calcined Dragon Bone 30g, Calcined Concha Osterae (*Ostrea gigas* Thunberg) 30g, Concha Margaritifear (*Pteria martensii* (Dunker)) 30g, Calcined Magentitum 30g, Petiole charcoal of *Trachycarpus fortunei* (Hook). H. Wendi. 30g, Root charcoal of *Sanguisorba officinalis* L. 30g, Root of *Angelica sinensis* (Oliv.) Diels. 10g, Processing Root of *Rehmannia glutinosa* (Gaertn.) DC.10g, [Beijing Tcmages Pharmaceutical Co., LTD]	200 ml bid	Y- Prepared according to ChP; Pharmacy preparation
	Blood stasis syndrome (37/20)	Calming the mind and relieving itching (Blood stasis)	No.2 Calm-the-Mind-and-Relieve-Itching Formula	Calcined Dragon Bone 30g, Calcined Concha Osterae (*Ostrea gigas* Thunberg) 30g, Concha Margaritifear (*Pteria martensii* (Dunker)) 30g, Calcined Magentitum 30g, Petiole charcoal of *Trachycarpus fortunei* (Hook). H. Wendi. 30g, Root charcoal of *Sanguisorba officinalis* L. 30g, Rhizome of *Sparganium stoloniferum* (Graebn.) Buch-Ham. 10g, Rhizome of *Curcuma zedoaria* (Christm.) Roscoe 10g, [Beijing Tcmages Pharmaceutical Co., LTD]	200 ml bid	
Lv (2018)	NR	NR	PSORI-CM02	Rhizome of *Curcuma zedoaria* (Christm.) Roscoe, Root of *Paeonia veitchii* Lynch, Rhizome of *Smilax glabra* Roxb, Whole plant of *Sarcandra glabra* (Thunb.) Nakai, Fruit of *Prunus mume* (Siebold) Siebold & Zucc.	1 pack bid	NR
Mao et al. (2019)	Blood heat syndrome (30/20)	Cooling blood and promoting blood circulation	Liang xue huo xue decoction (LXHXD)	Root of *Rehmannia glutinosa* (Gaertn.) DC. 30 g, Flower of *Sophora japonica* L. 30 g, Root and rhizome of *Salvia miltiorrhiza* Bunge 15 g, Rhizome of *Imperata cylindrica* (L.) Beauv. 30 g, Root of *Lithospermum erythrorhizon* Sieb. et Zucc. 15 g, Root of Paeonia veitchii Lynch 15 g, and dried lianoid stem of *Spatholobus suberectus* Dunn 30 g.	200 ml bid	NR
Zhang (2018)	Blood heat syndrome (3/4) Blood stasis syndrome (0/1) Blood dryness syndrome (37/35)	Promoting blood circulation, removing blood stasis and removing spots, removing wind, dampness and itching	PSORI-CM01	Root of *Paeonia veitchii* Lynch, Rhizome of *Curcuma zedoaria* (Christm.) Roscoe, Whole plant of *Sarcandra glabra* (Thunb.) Nakai, Root and rhizome of *Glycyrrhiza uralensis* Fisch. ex DC, Fruit of *Prunus mume* (Siebold) Siebold & Zucc., Root of *Lithospermum erythrorhizon* Sieb. et Zucc., Rhizome of *Smilax glabra* Roxb, [Jiangyin Tianjiang Pharmaceutical Co., Ltd]	5.5 g bid	Y - Prepared according to ChP; Pharmacy preparation
Li et al. (2020a)	NR	tonifying the kidney and removing obstruction in the channels on the blood	Kunxian capsule	*Tripterygium hypoglaucum* (Levl.) Hutch, Leaf of *Epimedium brevicornum* Maxim., Fruit of *Lycium barbarum* L, Seed of *Cuscuta chinensis* Lam., [Guangzhou chenliji Pharmaceutical Co., Ltd]	0.3 g*2# tid	Y - Prepared according to ChP YBZ07522006

C, control group; E, experimental group; NR, no report; AEs, adverse events; RER, recurrence rate; DLQI, dermatology life quality index; VAS, visual analog scale; M, Male; F, Female; w, weeks; m, months; y, years.

ChP, Pharmacopoeia of the People's Republic of China; GPHCM, Guangdong Provincial Hospital of Chinese Medicine; NR, Not Remined; bid, bis in die; tid, ter in die; YBZ07522006, State food and drug administration of the People’s Republic of China, NO. YBZ07522006-2009Z.

### Description of the Chinese Herbal Medicines

Fifty-four herbs were included in the 11 studies. The top eight most frequently used herbs were used more than 5 times and included the following: Rhizoma Smilacis Glabrae (rhizome of *Smilax glabra* Roxb), Radix Paeoniae Rubra (root of *Paeonia veitchii* Lynch), Rhizoma Curcumae Aeruginosae [rhizome of *Curcuma zedoaria* (Christm.) Roscoe], Radix Salviae Miltiorrhizae (root and rhizome of *Salvia miltiorrhiza* Bunge), Radix Rehmanniae [root of *Rehmannia glutinosa* (Gaertn.) DC], Caulis Spatholobi (dried lianoid stem of *Spatholobus suberectus* Dunn), Radix Arnebiae (root of *Lithospermum erythrorhizon* Sieb. et Zucc.), Chinese angelica [root of *Angelica sinensis* (Oliv.) Diels.] (Additional File: Supplementary Table S3).

### Risk of Bias

The methodological quality of each study was assessed with the Jadad score and all the included trials appeared to be of high quality, with a Jadad score between 4 and 7. Two trials failed (Wang, 2003; Mao et al., 2019) to report the concealment method, one trial (Li B. Y. et al., 2020) used a randomized method based on the order of patients' visits, two trials (Wang, 2003; Mao et al., 2019) did not describe the method of randomization. More details are shown in Table 2.

**TABLE 2:** Jadad scale of the included trials.

Author Year	Jadad Scale				
	a	b	c	d	T
Wang (2003)	1	0	2	1	4
Zhou (2011)	2	2	2	1	7
Zhou (2012)	2	2	2	1	7
Chen (2016)	2	2	2	1	7
Yao et al. (2016)	2	2	2	1	7
Li et al. (2017)	2	2	2	1	7
She (2017)	2	2	2	1	7
Lv (2018)	2	2	2	1	7
Mao et al. (2019)	1	1	2	1	5
Zhang (2018)	2	2	2	1	7
Li B. Y. et al. (2020)	1	2	2	1	6

From a to d, dimension of the Jadad scale. Points awarded: a, study was described as randomized, 1 point; the method was appropriate (table of random numbers, computer generated, etc.), 2 points; b, study used allocation concealment, 1 point; the method was appropriate (taken by the third one who wasn’t researcher or patient, opaque envelope, etc.), 2 points; c, study was described as double blind, 1 point; the method was appropriate (identical placebo, active placebo, dummy, etc.), 2 points; d, study reported withdraws and dropouts and described the reasons. T, total.

## Primary Outcomes

### PASI Score

**FIGURE 2:**
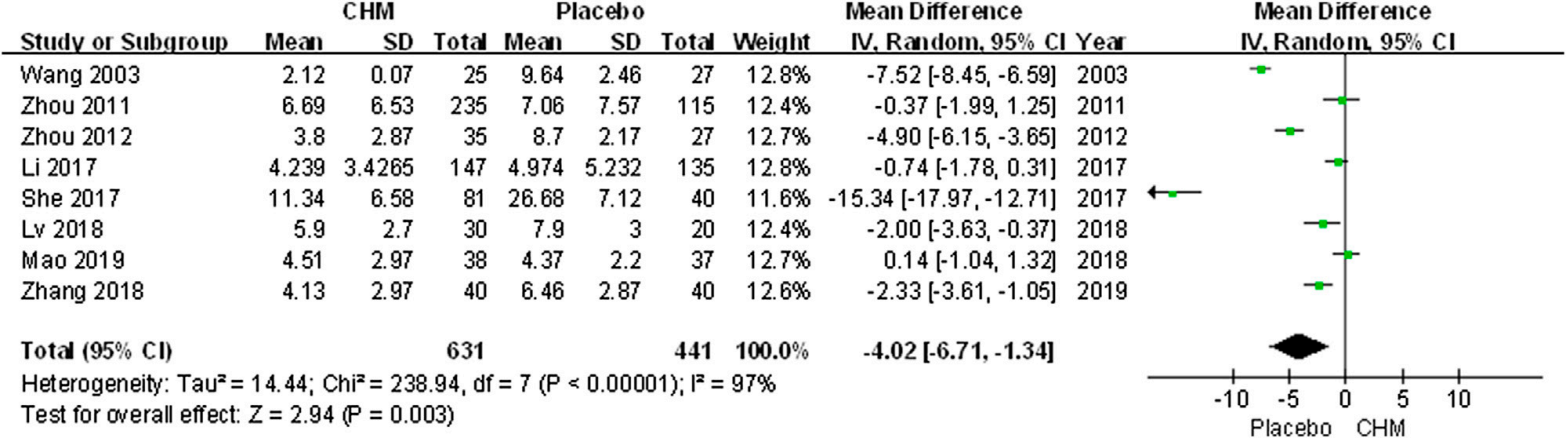
Forest plot of PASI Score between CHM and placebo groups. (CHM: Chinese Herbal Medicine; PASI: psoriasis area severity index).

**FIGURE 3:**
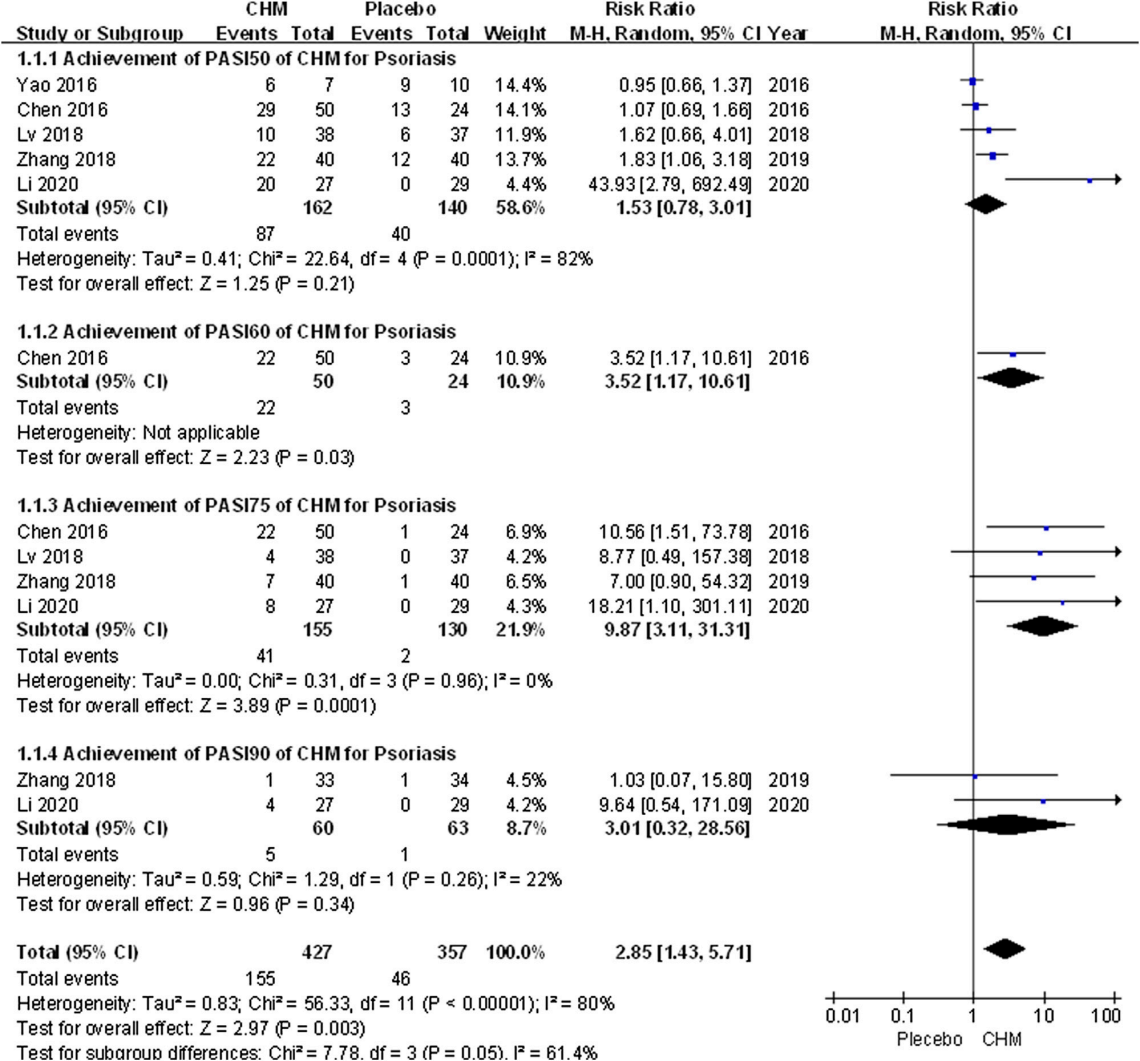
Forest plot of achievement of PASI-50, PASI-60, PASI-75 and PASI-90between CHM and placebo group. (CHM: Chinese Herbal Medicine; PAIS-50/60/75/90: PASI score decreases more than 50%/60%/75%/90% from baseline).

We conducted a comprehensive analysis of the PASI scores recorded in eight trials. The PASI score of the CHM group was significantly lower than that of the placebo group after treatment (MD, −4.02; 95% CI, −6.71 to −1.34; *p* = 0.003) (Figure 2). Additionally, we performed a subgroup analysis based on different PASI reduction indices. For PASI-50 and PASI-90, there were no statistically significant result (PASI-50: RR, 1.53; 95% CI, 0.78 to 3.01; *p* = 0.21; PASI-90: RR, 3.01; 95% CI, 0.32 to 28.56; *p* = 0.34). Meanwhile, to achieve PASI-60 and PASI-75, the arrival rate of the CHM group was higher than that of the placebo group (PASI-60: RR, 3.52; 95% CI, 1.17 to 10.61; *p* = 0.03; PASI-75: RR, 9.87; 95% CI, 3.11 to 31.31; *p* = 0.0001) (Figure 3).

## Secondary Outcome

### Efficacy Rate

**FIGURE 4:**
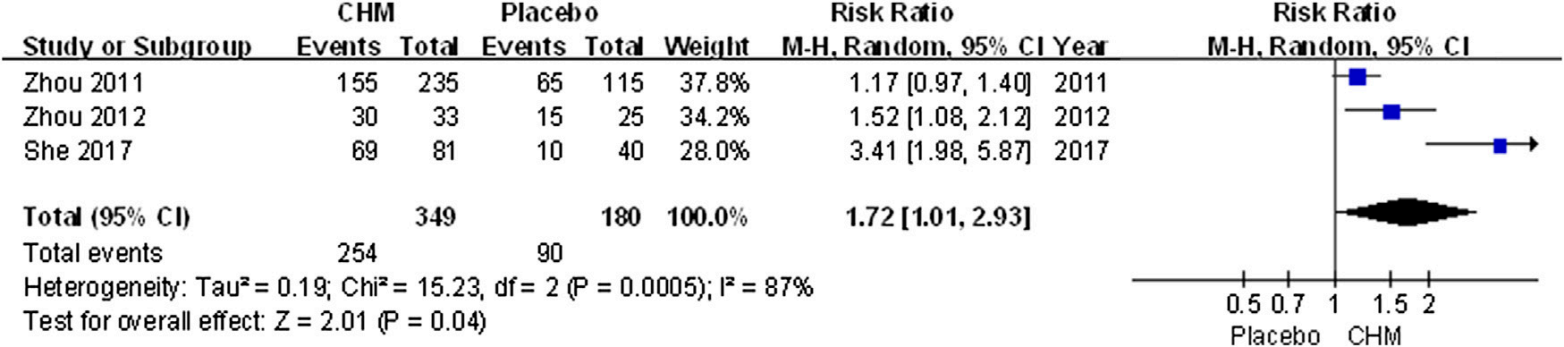
Forest plot of efficacy rate between CHM and placebo groups. (CHM: Chinese Herbal Medicine).

The efficacy rate was higher in patients receiving CHM than those receiving placebo (RR, 1.72; 95% CI, 1.01 to 2.93; *p* = 0.04) (Figure 4).

### Dermatology Life Quality Index

**FIGURE 5:**
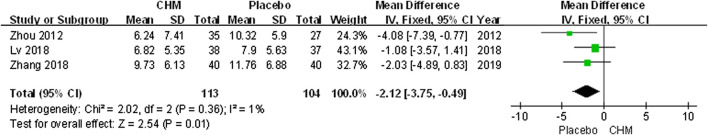
Forest plot of dermatology life quality index (DLQI) score between CHM and placebo group. (CHM: Chinese Herbal Medicine; DLQI: dermatology life quality index).

Three trials applied the DLQI to assess the quality of life of patients with psoriasis. The comprehensive results suggested a greater impact of CHM than placebo (MD, −2.12; 95% CI, −3.75 to −0.49; *p* = 0.01) (Figure 5).

### Pruritus Severity

**FIGURE 6:**
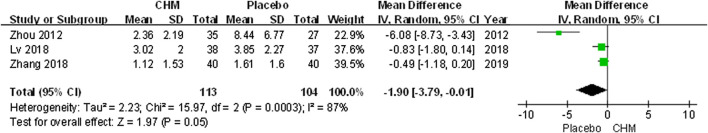
Forest plot of VAS score between CHM and placebo group. (CHM: Chinese Herbal Medicine; VAS: visual analog scale).

The visual analogue scale (VAS) was performed to assess pruritus severity. Our meta-analysis indicated that there was no significant difference between the CHM group and the control group (MD, −1.90; 95% CI, −3.79 to −0.01; *p* = 0.05) (Figure 6).

### Recurrence Rate

**FIGURE 7:**
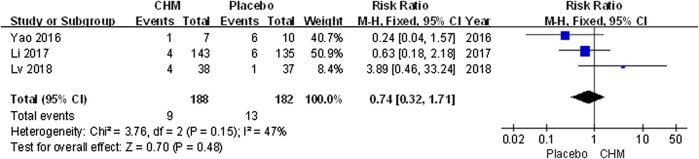
Forest plot of recurrence rate between CHM and placebo group. (CHM: Chinese Herbal Medicine).

Only three trials (Yao et al., 2016; Li et al., 2017; Lv, 2018) included follow-up surveys and assessed recurrence rate. The meta-analysis revealed that the recurrence rate associated with CHM was similar that with a placebo (RR, 0.74; 95% CI, 0.32 to 1.71; *p* = 0.48) (Figure 7).

### Safety

**FIGURE 8:**
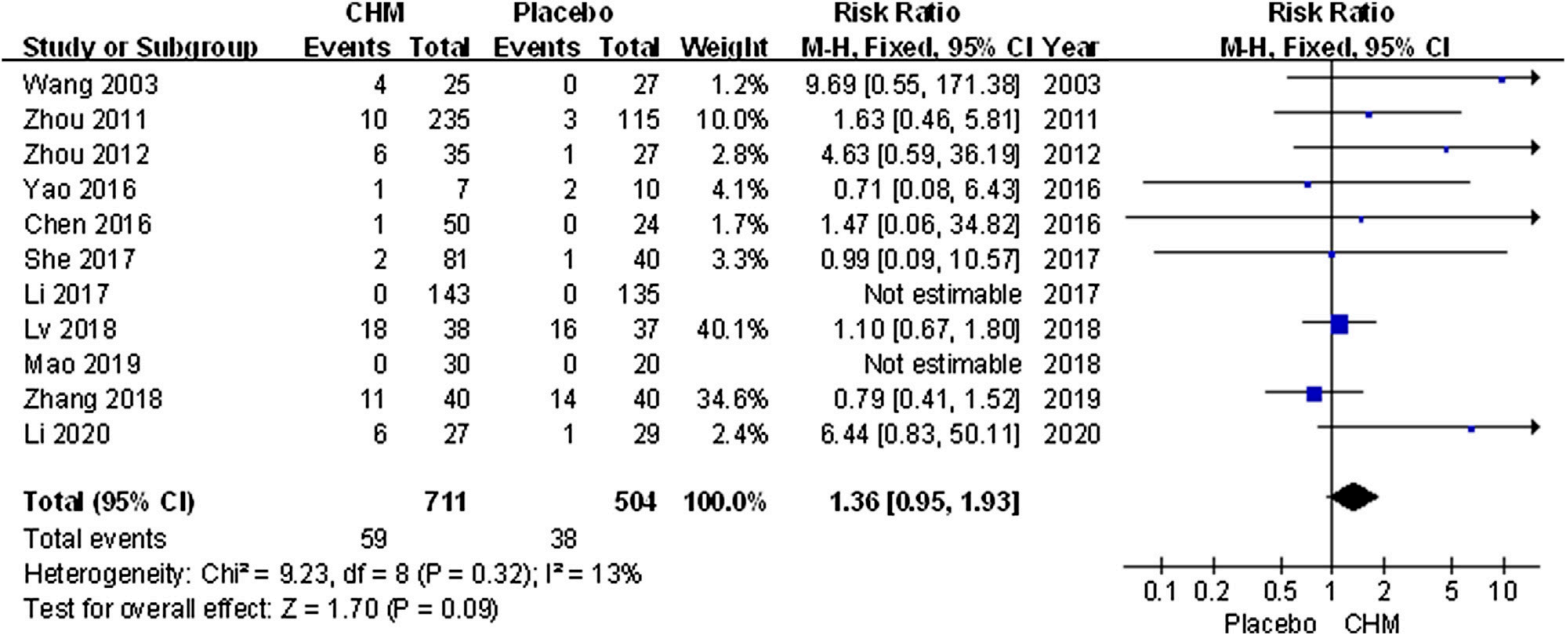
Forest plot of adverse events between CHM and placebo groups. (CHM: Chinese Herbal Medicine).

All trials assessed AEs including infection, gastrointestinal discomfort, abnormal liver function, limb erythema, and burning heat sensation. Meta-analysis results showed that the incidence of AEs in patients treated with CHM was similar to that of the placebo (RR, 1.36; 95% CI, 0.95 to 1.93; *p* = 0.09) (Figure 8).

## Discussion

This systematic review involved 11 randomized controlled trials that evaluated the efficacy and safety of CHM therapy in the treatment of psoriasis. The comprehensive results showed that the PASI score of the CHM group was significantly lower than of the placebo group, while the efficacy rate was higher in the CHM group than in the placebo group. To achieve PASI-50 and PASI-90, the arrival rate of the CHM group was similar to that of the placebo group; however, it was significantly improved for PASI-60 and PASI-75. The results suggested that there may be limited cure of the skin lesions. Indeed, the Chinese guidelines for the diagnosis and treatment of psoriasis (2018 version) pointed out that patients with mild-to-moderate psoriasis are mainly treated with internal CHM treatment, while the severe, pustular, erythrodermic, and arthropathic psoriasis types should be treated with integrated traditional Chinese and western medicine (Psoriasis Professional Committee of Dermatology and venereology Branch of Chinese Medical Association, 2019).

Furthermore, the comprehensive results suggested a greater impact of CHM on the DLQI of patients. On the contrary, there is currently no evidence regarding the efficacy of CHM in reducing episodes of psoriasis relapse and relief pruritus severity. In terms of safety, there was no significant difference between the CHM group and the control group, which indicated that CHM can be safely used for psoriasis.

Psoriasis is a chronic, relapsing skin disease that has psychological and physical effects and substantially impacts the quality of life of patients (Zandi et al., 2011). The most common approach to measure the health-related quality of life (QOL) is psychometric instruments, which measure QOL in many fields; reason why the DLQI is used in dermatology. Our results suggested that the clearance of skin damage by traditional Chinese medicine is effective and safe, and is closely related to improving the quality of life of patients. In addition, the integration of CHM and western medicine has been proven effective. It is known that the treatment of psoriasis in combination with a local sequential therapy can be used to eliminate skin lesions, as well as to relieve itching faster and prolong the therapeutic effect (Yao et al., 2016). Coincidentally, patients who received both a traditional Chinese medicine bath and NB-UVB treatment had a longer remission period (Wang et al, 2020). A systematic review found that oral Chinese medicine was neither better nor inferior to acitretin, which may reduce the common adverse reactions of acitretin. (Zhang et al, 2015).

The 11 studies included 54 herbs, of which the eight most used herbs include the following: Rhizoma Smilacis Glabrae (rhizome of *Smilax glabra* Roxb), Radix Paeoniae Rubra (root of *Paeonia veitchii* Lynch), Rhizoma Curcumae Aeruginosae (rhizome of *Curcuma zedoaria* [Christm.] Roscoe), Radix Salviae Miltiorrhizae (root and rhizome *of Salvia miltiorrhiza* Bunge), Radix Rehmanniae (root of *Rehmannia glutinosa* [Gaertn.] DC.), Caulis Spatholobi (dried lianoid stem of *Spatholobus suberectus* Dunn), Radix Arnebiae (root of *Lithospermum erythrorhizon* Sieb. et Zucc.) and Chinese angelica (root of *Angelica sinensis* [Oliv.] Diels.). Generally, the purpose of these traditional Chinese medicines included cooling, activating, and nourishing the blood. The ongoing high-quality randomized controlled double-blind clinical trials are shown in Table 3, which indicated that it is worthy of being investigated to promote clinical application. Pharmacological studies have demonstrated the mechanism of some drugs in the treatment of psoriasis. For instance, Rhizoma smilacis glabrae plays an anti-inflammatory and immunomodulatory role by inhibiting overactivated macrophages and regulating the activity of T lymphocytes (Jiang and Xu, 2003). The main bioactive components of Rhizoma smilacis glabrae, including astilbin, neoastilbin, neoisoastilbin, isoastilbin, engeletin, and isoengeletin are all flavonoids (Zhang et al., 2019). Tanshinone IIA, the effective component of Radix salviae miltiorrhizae, can inhibit the proliferation of KC, induce apoptosis, and block the cell cycle of KC (Li F. L. et al., 2012). Radix arnebiae can be used both internally and externally to treat psoriasis, which decreases IL-17-induced VEGF expression by the inhibition of JAK2/STAT3 signaling and exerts an anti-inflammatory effect via proteasome inhibition (Lu et al., 2011; Xu et al., 2014). Radix paeoniae rubra can inhibit the upregulation of pro-inflammatory mediators, such as tumor necrosis factor-α and IL-1 β (Guo et al., 2012). Pharmacological studies have proved that Caulis spatholobi has the effect of increasing the expression of caspase-3 and inhibiting human neutrophil elastase activity (Ha et al., 2004; Huang et al., 2013). These results demonstrate that the anti-inflammatory and immunomodulatory effects, and inhibition of epidermal cell proliferation by CHM contribute to its efficacy for psoriasis treatment.

**TABLE 3:** Chinese Herbal Medicine for Psoriasis in clinical development.

Title	Condition or desease	Intervention (E/C)	Status	Phase	Identifier
Oral chinese herbal medicine for Psoriasis Vulgaris with blood heat syndrome	Psoriasis Vulgaris	Jueyin granules/Jueyin granules Placebo	Not yet recruiting	Phase 2	NCT03961230
YXBCM01(PSORI-CM01) granules for psoriasis vulgaris: a Randomised double-blind, double-dummy, controlled clinical study	Plaque Psoriasis	PSORI-CM01 (YXBCM01) granule/placebo	Terminated	Not applicable	NCT02153840
Oral chinese herbal medicine for Psoriasis Vulgaris with blood stasis syndrome	Psoriasis Vulgaris	Taodan Granules/Taodan granules Placebo	Not yet recruiting	Phase 2	NCT03942198
A clinical trial of topical application containing Radix Rubiae for plaque-type psoriasis	Plaque Psoriasis	Topical application containing Radix Rubiae/vehicle preparation	Not yet recruiting	Phase 1	ChiCTR-IPC-15007019
Treatment of Kunxian capsule for moderate to severe plaque psoriasis: a multi-center, randomized, double-blinded, double-dummy, positive-drug, parallel-controlled clinical trial	Plaque Psoriasis	Kunxian Capsule + Methotrexate tablets placebo/Kunxian Capsule placebo + Methotrexate tablets	Unknown	Phase 4	ChiCTR1900025165
A randomized double-blind placebo parallel control trial for Chinese herbal bath in the treatment of psoriasis vulgaris (blood heat syndrome)	Psoriasis vulgaris	Basic treatment + Chinese herbal bath therapy/Basic treatment + Chinese herbal placebo bath therapy	Not yet recruiting	Not applicable	ChiCTR2000037510
A multicenter, randomized, double-blind, placebo-controlled study based on cooling blood latent Yang method combined with biological agents on the impact in recurrence of plaque psoriasis	Plaque Psoriasis	Izituzumab + JYKL (4 weeks) + JYKL (8 weeks)/Izituzumab + JYKL (4 weeks) + JYKL placebo (8 weeks)/Izituzumab + JYKL placebo (4 weeks) + JYKL (8 weeks)/Izituzumab + JYKL placebo (4 weeks) + JYKL placebo (8 weeks)	Not yet recruiting	Not applicable	ChiCTR2000036609
A Randomized Controlled study on the Combined and sequential optimal plan of traditional chinese medicine internal and external therapy for Psoriasis	Mild and moderate plaque psoriasis	Jueyin granules + moving cupping placebo therapy/Jueyin granules placebo + moving cupping therapy	Not yet recruiting	Phase 4	ChiCTR2000035992
Oral Chinese herbal medicine (Guben Huayu Formula) concurrent with methotrexate for patients with moderate-to-severe psoriasis: a Randomized controlled trial	Moderate to severe Psoriasis	Gu ben hua yu fang plus methotrexate/Chinese herbal medicine placebo plus methotrexate	Recruiting	Not applicable	ChiCTR2000035278

This systematic review has some limitations. First, since we take high-quality RCT research studies as the starting point, satisfying trials with high quality are limited. Moreover, there are no high-quality double-blind placebo RCTs on CHM and Western medicine to better evaluate the efficacy and advantages of CHM. Second, only three trials (Yao et al., 2016; Li et al., 2017; Mao et al., 2019) had been registered in the Clinical Trials Registry Platform. Therefore, it is not possible to use the protocol to confirm no selective reporting. Third, although the methodological quality of the included trials was generally high based on the Jadad scale, there were still some methodological defects. Three (Wang, 2003; Mao et al., 2019; Li B. Y. et al., 2020) trials failed to report the specific randomized method, two trials failed to (Wang, 2003; Mao et al., 2019) report the concealment method, and only three trials (Yao et al., 2016; Li et al., 2017; Lv, 2018) reported the follow-up data. Therefore, the results of these studies should be interpreted carefully. Finally, it was difficult to unify the drug composition, doses and the course of treatment in the included trials. This may affect the validity of the research results. A large high-quality RCT with sufficient standardized information on the quality control, content, usage, and course of treatment of CHM should be conducted in future studies (Gagnier et al., 2006).

## Conclusion

In summary, CHM appears safe and effective in treating patients with psoriasis and has a great impact in improving their quality of life, but does not lead to complete elimination of skin lesions and improvement in pruritus severity. No evidence is available on the ability of CHM to reduce the relapse the rate of psoriasis. All the included studies reported adverse events; overall, CHM was safe for the treatment of psoriasis.

## Data Availability

The original contributions presented in the study are included in the article/Supplementary Material, further inquiries can be directed to the corresponding authors.

## Abbreviation

AEs, adverse events; CENTRAL, Cochrane Central Registry of Controlled Trials; ChiCTR: Chinese Clinical Trial Registry; CHM, Chinese herbal medicine; CNKI, China Network Knowledge Infrastructure; CQVIP, China Science and Technology Journal Database; DLQI, dermatology life quality index; IL-17, interleukin-17; IL-6, interleukin-6; IL-1β, interleukin-1β; IFN-γ, Interferon-γ; KC, keratinocytes; MeSH, medical subject headings; PASI, psoriasis area severity index; PASI-50/60/75/90, PASI score decreases more than 50%/60%/75%/90% from baseline; PRISMA, preferred reporting items for systematic review and meta-analyses; RCTs, randomized control trials; RER, recurrence rate; TNF-α, tumor necrosis factor-α; VAS, visual analog scale; VEGF, vascular endothelial growth factor.
