# Erratum: Chinese Herbal Medicine for Psoriasis: Evidence from 11 High-Quality Randomized Controlled Trials

**DOI:** 10.3389/fphar.2021.672760

**Published:** 2021-03-31

**Authors:** Frontiers Production Office

**Affiliations:** Frontiers Media SA, Lausanne, Switzerland

**Keywords:** Chinese herbal medicine, psoriasis, high-quality, randomized controlled trials, meta-analysis

Due to a production error, there was a mistake in Table 1 as published. The table was incomplete as a section inside was mistakenly omitted. The corrected Table 1 appears below. The publisher apologizes for this mistake.

**TABLE 1:** Summary of the characteristics of the included trails.

Author Year	Sample Size		Age (years) (Mean ± SD)		Gender (M/F)		Duration of psoriasis (Mean ± SD)		Intervention		Course of treatment	Adverse events		0utcome
	E	C	E	C	E	C	E	C	E	C		E	C	
Wang (2003)	25	27	26.68 ± 12.3	28.62 ± 14.23	16/9	20/7	56.68 ± 40.23 m	61.24 ± 39.12 m	CHM Capsule	Placebo	4w	4	0	PASI, efficiency, AEs
Zhou (2011)	235	115	38.13 ± 12.47	39.50 ± 13.07	146/89	74/41	NR	NR	CHM decoction	Placebo	8W	10	2	PASI, efficiency, AEs
Zhou (2012)	35	27	34.11 ± 9.15	31.04 ± 7.88	16/19	11/16	3.97 ± 5.92 y	3.70 ± 5.78 y	CHM decoction	Placebo	8W	6	1	PASI, DLQI, VAS, efficiency, AEs
Chen (2016)	50	24	39.5 ± 12.45	36.3 ± 11.38	36/14	17/7	15.6 ± 10.83 y	15.2 ± 8.56y	CHM decoction	Placebo	8W	1	0	PASI 50, PASI 60, PASI 75, AEs
Yao et al. (2016)	8	10	45.43 ± 11.84	41.60 ± 13.24	7/0	8/2	144.57 ± 73.77 m	86.20 ± 54.02 m	CHM decoction + topical sequential treatment	Placebo + topical sequential treatment	12W	1	2	PASI50, RER, AEs
Li et al. (2017)	143	135	40 ± 13	36 ± 12	78/65	74/61	NR	NR	CHM Ointment	Placebo	4W	0	0	PASI, RER, AEs
She (2017)	81	40	30.12 ± 6.31	28.97 ± 5.03	41/40	21/19	11.87 ± 4.49 y	10.94 ± 5.38 y	CHM Particle	Placebo	12W	2	1	PASI, efficiency, AEs
Lv (2018)	38	37	42.55 ± 10.86	38.62 ± 13.65	34/4	31/6	9.68 ± 6.04 y	9.78 ± 6.31 y	CHM Particle	Placebo	12W	18	16	PASI, PASI50, PASI 75, DLQI, VAS, RER, AEs
Mao et al. (2019)	30	20	28.13 ± 9.71	31.50 ± 10.13	18/12	13/7	32.7 ± 9.3 m	36.5 ± 8.7 m	CHM decoction	Placebo	6W	0	0	PASI, AEs
Zhang (2018)	40	40	40.73 ± 10.90	43.50 ± 11.75	33/7	31/9	NR	NR	CHM Particle	Placebo	4W	11	14	PASI, PASI50, PASI75, PASI90, DLQI, VAS, AEs
Li et al. (2020a)	27	29	37.74 ± 10.50	41.45 ± 14.40	16/11	17/12	NR	NR	CHM Capsule	Placebo	4W	6	1	PASI50, PSAI75, PASI90, AEs

Author Year	TCM Syndrome (E/C)	Curative effect	Name of herbs	Compositions (Species, source, concentration)	Usage	Quality control reported? (Y/N)
Wang (2003)	NR	Clearing heat and detoxifying, removing blood stasis and removing spots, removing wind, dampness and itching	Compound Indigo Naturalis Capsules	Powder of leaves of *Strobilanthes cusia* (Nees) Kuntze, Whole plant of *Portulaca oleracea* L., Rhizome of *Smilax glabr*a Roxb, Root bark of *Dictamnus dasycarpus* Turcz., Root of *Angelica dahurica* var. formosana (H.Boissieu) Yen, Root of *Lithospermum erythrorhizon* Sieb. et Zucc., Rhizome of *Dryopteris crassirhizoma* Nakai, Whole plant of *Taraxacum mongolicum* Hand-Mazz., Root and rhizome of Salvia miltiorrhiza Bunge, Root and rhizome of *Dioscorea collettii* var. hypoglauca (Palib.) S.J.Pei & C.T.Ting, Fruit of *Prunus mume* (Siebold) Siebold & Zucc., Fruit of *Schisandra chinensis* (Turcz.) Baill., Fruit of Crataegus pinnatifida Bunge, Massa medicata fermentata	0.5 g*4 tid	Y-repared according to ChP Z20010157
Zhou (2011)	Blood heat syndrome (78/39)	Cooling blood and detoxification	Cooling Blood and detoxification decoction	Rhizome of *Smilax glabra* Roxb 30g, Flower of *Sophora japonica* L. 15g, Root of *Lithospermum erythrorhizon* Sieb. et Zucc. 10g, Rhizome of *Paris polyphylla* 9g, Root of *Rehmannia glutinosa* (Gaertn.) DC. 15g, Root bark of *Dictamnus dasycarpus* Turcz. 10g, Root of *Paeonia veitchii* Lynch 10g, [Pharmacy Department of Beijing Hospital of traditional Chinese Medicine]	100 ml bid	Y- Prepared according to ChP; Hospital preparation
	Blood dryness syndrome (79/38)	Nourishing blood and detoxification	Nourishing Blood and detoxification decoction	Root and rhizome of *Salvia miltiorrhiza Bunge* 15g, Root of *Angelica sinensis* (Oliv.) Diels. 15g, Root of *Rehmannia glutinosa* (Gaertn.) DC. 15g, Root of *Ophiopogon japonicus* (L. f.) Ker-Gawl. 10g, Root of *Scrophularia ningpoensis* Hemsl. 15g, dried lianoid stem of *Spatholobus suberectus* Dunn 15g, Rhizome of *Smilax glabra* Roxb 30g, [Pharmacy Department of Beijing Hospital of Traditional Chinese Medicine]	100 ml bid	
	Blood stasis syndrome (78/38)	Promoting blood circulation and detoxification	Promoting Blood Circulation and detoxification decoction	Whole grass of *Hedyotis diffusa* Willd. 30g, Rhizome of *Curcuma zedoaria* (Christm.) Roscoe 10g, Wing-shaped cork of *Euonymus alatus* (Thunb.) Siebold 10g, Flower of *Carthamus tinctorius* L. 10g, dried lianoid stem of *Spatholobus suberectus* Dunn 30g, Kernel of *Amygdalus persica L.* 10g, Root and rhizome of *Salvia miltiorrhiza* Bunge 15g, [Pharmacy Department of Beijing Hospital of traditional Chinese Medicine]	100 ml bid	
Zhou (2012)	Blood heat syndrome (35/27)	Cooling blood and promoting blood circulation	Liangxuehuoxue Complex Prescription	Leaf of *Isatis tinctoria L.* 15g, Root of *Rehmannia glutinosa* (Gaertn.) DC. 30g, Root of *Scutellaria baicalensis Georgi* 12g, Root of *Lithospermum erythrorhizon* Sieb. et Zucc. 9g, Root and rhizome of *Salvia miltiorrhiza* Bunge 12g, Root of *Paeonia veitchii* Lynch 6g, Root bark of *Paeonia suffruticosa* Andr. 9g, Root of *Angelica sinensis* (Oliv.) Diels. 12g, Rhizome of *Smilax glabra* Roxb 30g	100 ml bid	NR
Chen (2016)	Blood heat syndrome (15/10)	Cooling blood and detoxification	Cooling Blood and detoxification decoction	Rhizome of *Smilax glabra* Roxb, Flower of *Sophora japonica* L., Root of *Lithospermum erythrorhizon* Sieb. et Zucc., Root of *Rehmannia glutinosa* (Gaertn.) DC., Root of *Paeonia veitchii* Lynch, Root bark of *Paeonia suffruticosa* Andr., Rhizome of *Imperata cylindrica* (L.) Beauv., Root bark of *Dictamnus dasycarpus* Turcz., Root of *Saposhnikovia divaricata* (Turcz. ex Ledeb.) Schischk., Whole grass of *Hedyotis diffusa* Willd., Rhizome of *Paris polyphylla*, Root of *Isatis tinctoria L.*, [Beijing Institute of traditional Chinese Medicine]	200 ml	Y- Prepared according to ChP; Hospital preparation
	Blood dryness syndrome (17/5)	Nourishing blood and detoxification	Nourishing Blood and detoxification decoction	Root of *Angelica sinensis* (Oliv.) Diels., Root of *Rehmannia glutinosa* (Gaertn.) DC., Root and rhizome of *Salvia miltiorrhiza* Bunge, dried lianoid stem of *Spatholobus suberectus* Dunn, Root of *Ophiopogon japonicus* (L. f.) Ker-Gawl., Root of *Scrophularia ningpoensis* Hemsl., Root of Trichosanthes kirilowii Maxim., Rhizome of *Smilax glabra* Roxb, Whole grass of *Hedyotis diffusa* Willd., Rhizome of *Paris polyphylla*, Rhizome of *Atractylodes lancea* (Thunb.) DC., Fruit of *Tribulus terrestris* L., [Beijing Institute of traditional Chinese Medicine]	200 ml	
	Blood stasis syndrome (4/3)	Promoting blood circulation and detoxification	Promoting Blood Circulation and detoxification decoction	Root of *Angelica sinensis* (Oliv.) Diels., Rhizome of *Curcuma zedoaria* (Christm.) Roscoe, Root and rhizome of *Salvia miltiorrhiza* Bunge dried lianoid stem of *Spatholobus suberectus* Dunn, Kernel of *Amygdalus persica L.*, Root of *Scrophularia ningpoensis* Hemsl., Root and rhizome of *Clematis chinensis* Osbeck, Branch of *Cinnamomum cassia* (L.) J. Presl, Rhizome of *Atractylodes lancea* (Thunb.) DC., Wing-shaped cork of *Euonymus alatus* (Thunb.) Siebold, Whole grass of *Hedyotis diffusa* Willd., Rhizome of *Paris polyphylla* [Beijing Institute of traditional Chinese Medicine]	200 ml	
Yao et al. (2016)	NR	Promoting blood circulation, removing blood stasis and removing spots, removing wind, dampness and itching	PSORI-CM01	Root of *Paeonia veitchii* Lynch, Rhizome of *Curcuma zedoaria* (Christm.) Roscoe, Whole plant of *Sarcandra glabra* (Thunb.) Nakai, Root and rhizome of *Glycyrrhiza uralensis* Fisch. ex DC., Fruit of *Prunus mume* (Siebold) Siebold & Zucc., Root of *Lithospermum erythrorhizon* Sieb. et Zucc., and Rhizome of *Smilax glabra* Roxb, [Pharmaceutical department of Chinese herbal medicine of GPHCM]	100 ml bid	Y- Prepared according to ChP; Hospital preparation
Li et al. (2017)	Blood heat syndrome (143/135)	Clearing excess heat and dampness, and relieving swelling and pain.	Pulian Ointment	Powder of bark of *Phellodendron amurense* Rupr. 50g, Powder of root of *Scutellaria baicalensis* Georgi 50g and white petroleum jelly 400 g, [China-Japan Friendship Hospital]	2 mm larger than the skin lesion area bid	Y- Prepared according to ChP; Hospital preparation
She (2017)	Blood dryness syndrome (44/20)	Calming the mind and relieving itching (Blood dryness)	No.1 Calm-the-Mind-and-Relieve-Itching Formula	Calcined Dragon Bone 30g, Calcined Concha Osterae (*Ostrea gigas* Thunberg) 30g, Concha Margaritifear (*Pteria martensii* (Dunker)) 30g, Calcined Magentitum 30g, Petiole charcoal of *Trachycarpus fortunei* (Hook). H. Wendi. 30g, Root charcoal of *Sanguisorba officinalis* L. 30g, Root of *Angelica sinensis* (Oliv.) Diels. 10g, Processing Root of *Rehmannia glutinosa* (Gaertn.) DC.10g, [Beijing Tcmages Pharmaceutical Co., LTD]	200 ml bid	Y- Prepared according to ChP; Pharmacy preparation
	Blood stasis syndrome (37/20)	Calming the mind and relieving itching (Blood stasis)	No.2 Calm-the-Mind-and-Relieve-Itching Formula	Calcined Dragon Bone 30g, Calcined Concha Osterae (*Ostrea gigas* Thunberg) 30g, Concha Margaritifear (*Pteria martensii* (Dunker)) 30g, Calcined Magentitum 30g, Petiole charcoal of *Trachycarpus fortunei* (Hook). H. Wendi. 30g, Root charcoal of *Sanguisorba officinalis* L. 30g, Rhizome of *Sparganium stoloniferum* (Graebn.) Buch-Ham. 10g, Rhizome of *Curcuma zedoaria* (Christm.) Roscoe 10g, [Beijing Tcmages Pharmaceutical Co., LTD]	200 ml bid	
Lv (2018)	NR	NR	PSORI-CM02	Rhizome of *Curcuma zedoaria* (Christm.) Roscoe, Root of *Paeonia veitchii* Lynch, Rhizome of *Smilax glabra* Roxb, Whole plant of *Sarcandra glabra* (Thunb.) Nakai, Fruit of *Prunus mume* (Siebold) Siebold & Zucc.	1 pack bid	NR
Mao et al. (2019)	Blood heat syndrome (30/20)	Cooling blood and promoting blood circulation	Liang xue huo xue decoction (LXHXD)	Root of *Rehmannia glutinosa* (Gaertn.) DC. 30 g, Flower of *Sophora japonica* L. 30 g, Root and rhizome of *Salvia miltiorrhiza* Bunge 15 g, Rhizome of *Imperata cylindrica* (L.) Beauv. 30 g, Root of *Lithospermum erythrorhizon* Sieb. et Zucc. 15 g, Root of Paeonia veitchii Lynch 15 g, and dried lianoid stem of *Spatholobus suberectus* Dunn 30 g.	200 ml bid	NR
Zhang (2018)	Blood heat syndrome (3/4) Blood stasis syndrome (0/1) Blood dryness syndrome (37/35)	Promoting blood circulation, removing blood stasis and removing spots, removing wind, dampness and itching	PSORI-CM01	Root of *Paeonia veitchii* Lynch, Rhizome of *Curcuma zedoaria* (Christm.) Roscoe, Whole plant of *Sarcandra glabra* (Thunb.) Nakai, Root and rhizome of *Glycyrrhiza uralensis* Fisch. ex DC, Fruit of *Prunus mume* (Siebold) Siebold & Zucc., Root of *Lithospermum erythrorhizon* Sieb. et Zucc., Rhizome of *Smilax glabra* Roxb, [Jiangyin Tianjiang Pharmaceutical Co., Ltd]	5.5 g bid	Y - Prepared according to ChP; Pharmacy preparation
Li et al. (2020a)	NR	tonifying the kidney and removing obstruction in the channels on the blood	Kunxian capsule	*Tripterygium hypoglaucum* (Levl.) Hutch, Leaf of *Epimedium brevicornum* Maxim., Fruit of *Lycium barbarum* L, Seed of *Cuscuta chinensis* Lam., [Guangzhou chenliji Pharmaceutical Co., Ltd]	0.3 g*2# tid	Y - Prepared according to ChP YBZ07522006

C, control group; E, experimental group; NR, no report; AEs, adverse events; RER, recurrence rate; DLQI, dermatology life quality index; VAS, visual analog scale; M, Male; F, Female; w, weeks; m, months; y, years.

ChP, Pharmacopoeia of the People's Republic of China; GPHCM, Guangdong Provincial Hospital of Chinese Medicine; NR, Not Remined; bid, bis in die; tid, ter in die; YBZ07522006, State food and drug administration of the People’s Republic of China, NO. YBZ07522006-2009Z.

The original article has been updated.

