# Supplementary material for: Chinese Herbal Medicine for Psoriasis: Evidence From 11 High-Quality Randomized Controlled Trials
Source: Front Pharmacol. 2021 Jan 22;11:599433. doi: 10.3389/fphar.2020.599433 (PMC7862748; doi:10.3389/fphar.2020.599433)
Supplement: Supplementary file 1 [file datasheet1.docx]

# Supplementary Information

**Chinese herbal medicine for psoriasis: Evidence from 10 high-quality randomized controlled trials**

**Supplementary Table S1: checklist**

| **Section/topic** | **#** | **Checklist item** | **Reported on page #** |
| --- | --- | --- | --- |
| **TITLE** | | |  |
| Title | 1 | Identify the report as a systematic review, meta-analysis, or both. | **Page 1 (Title)** |
| **ABSTRACT** | | |  |
| Structured summary | 2 | Provide a structured summary including, as applicable: background; objectives; data sources; study eligibility criteria, participants, and interventions; study appraisal and synthesis methods; results; limitations; conclusions and implications of key findings; systematic review registration number. | **Pages 3-4 (Abstract)** |
| **INTRODUCTION** | | |  |
| Rationale | 3 | Describe the rationale for the review in the context of what is already known. | **Pages 6-8 (Introduction)** |
| Objectives | 4 | Provide an explicit statement of questions being addressed with reference to participants, interventions, comparisons, outcomes, and study design (PICOS). | **Pages 6-8 (Introduction)** |
| **METHODS** | | |  |
| Protocol and registration | 5 | Indicate if a review protocol exists, if and where it can be accessed (e.g., Web address), and, if available, provide registration information including registration number. | **Page 9 (methods)** |
| Eligibility criteria | 6 | Specify study characteristics (e.g., PICOS, length of follow-up) and report characteristics (e.g., years considered, language, publication status) used as criteria for eligibility, giving rationale. | **Page 9 (Search strategy)** |
| Information sources | 7 | Describe all information sources (e.g., databases with dates of coverage, contact with study authors to identify additional studies) in the search and date last searched. | **Page 9 (Search strategy)** |
| Search | 8 | Present full electronic search strategy for at least one database, including any limits used, such that it could be repeated. | **Page 9 (Search strategy)** |
| Study selection | 9 | State the process for selecting studies (i.e., screening, eligibility, included in systematic review, and, if applicable, included in the meta-analysis). | **Page 9 (Search strategy)** |
| Data collection process | 10 | Describe method of data extraction from reports (e.g., piloted forms, independently, in duplicate) and any processes for obtaining and confirming data from investigators. | **Page 9 (Search strategy)** |
| Data items | 11 | List and define all variables for which data were sought (e.g., PICOS, funding sources) and any assumptions and simplifications made. | **Page 10 (Data extraction)** |
| Risk of bias in individual studies | 12 | Describe methods used for assessing risk of bias of individual studies (including specification of whether this was done at the study or outcome level), and how this information is to be used in any data synthesis. | **Page 11 (****Risk of bias assessments)** |
| Summary measures | 13 | State the principal summary measures (e.g., risk ratio, difference in means). | **Page 11 (****Statistical analysis)** |
| Synthesis of results | 14 | Describe the methods of handling data and combining results of studies, if done, including measures of consistency (e.g., I^2^) for each meta-analysis. | **Page 11 (Statistical analysis)** |
| **Section/topic** | **#** | **Checklist item** | **Reported on page #** |
| Risk of bias across studies | 15 | Specify any assessment of risk of bias that may affect the cumulative evidence (e.g., publication bias, selective reporting within studies). | **Page 11 (Risk of bias assessments)** |
| Additional analyses | 16 | Describe methods of additional analyses (e.g., sensitivity or subgroup analyses, meta-regression), if done, indicating which were pre-specified. | **Page 11 (****Statistical analysis)** |
| **RESULTS** | | |  |
| Study selection | 17 | Give numbers of studies screened, assessed for eligibility, and included in the review, with reasons for exclusions at each stage, ideally with a flow diagram. | **Pages 11-12 (Study selection of included studies)** |
| Study characteristics | 18 | For each study, present characteristics for which data were extracted (e.g., study size, PICOS, follow-up period) and provide the citations. | **Pages 12-13 (characteristics of included studies)** |
| Risk of bias within studies | 19 | Present data on risk of bias of each study and, if available, any outcome level assessment (see item 12). | **Pages 13-14 (Risk of bias assessments)** |
| Results of individual studies | 20 | For all outcomes considered (benefits or harms), present, for each study: (a) simple summary data for each intervention group (b) effect estimates and confidence intervals, ideally with a forest plot. | **Pages 14-15 (Primary outcomes / Secondary outcomes)** |
| Synthesis of results | 21 | Present results of each meta-analysis done, including confidence intervals and measures of consistency. | **Pages 14-15 (****Primary outcomes / Secondary outcomes)** |
| Risk of bias across studies | 22 | Present results of any assessment of risk of bias across studies (see Item 15). | **Pages 13-14 (Risk of bias assessments)** |
| Additional analysis | 23 | Give results of additional analyses, if done (e.g., sensitivity or subgroup analyses, meta-regression [see Item 16]). | **Pages 14-15 (Primary outcomes / Secondary outcomes)** |
| **DISCUSSION** | | |  |
| Summary of evidence | 24 | Summarize the main findings including the strength of evidence for each main outcome; consider their relevance to key groups (e.g., healthcare providers, users, and policy makers). | **Pages 15-18 (Discussion)** |
| Limitations | 25 | Discuss limitations at study and outcome level (e.g., risk of bias), and at review-level (e.g., incomplete retrieval of identified research, reporting bias). | **Pages 18-19 (Limitations)** |
| Conclusions | 26 | Provide a general interpretation of the results in the context of other evidence, and implications for future research. | **Pages 19 (Conclusion)** |
| **FUNDING** | | |  |
| Funding | 27 | Describe sources of funding for the systematic review and other support (e.g., supply of data); role of funders for the systematic review. | **Page 20 (Funding)** |

PRISMA: Preferred Reporting Items for Systematic Reviews and Meta-Analyse

**Table S2: Search Strategy**

| PubMed | 1. | Psoriasis[Mesh] |
| --- | --- | --- |
|  | 2. | (Psoriases[Title/Abstract]) OR (Pustulosis of Palms[Title/Abstract] AND Soles[Title/Abstract]) OR (Pustulosis Palmaris et Plantaris[Title/Abstract]) OR (Palmoplantaris Pustulosis[Title/Abstract]) OR (Pustular Psoriasis of Palms[Title/Abstract] AND Soles[Title/Abstract]) |
|  | 3. | 1 or 2 |
|  | 4. | (Traditional Chinese Medicine[Title/Abstract]) OR (Chung I Hsueh[Title/Abstract]) OR (Hsueh, Chung I[Title/Abstract]) OR (Traditional Medicine, Chinese[Title/Abstract]) OR (Zhong Yi Xue[Title/Abstract]) OR (Chinese Traditional Medicine[Title/Abstract]) OR (Chinese Medicine, Traditional[Title/Abstract]) OR (Chinese Drugs, Plant[Title/Abstract]) OR (Chinese Herbal Drugs[Title/Abstract]) OR (Herbal Drugs, Chinese[Title/Abstract]) OR (Plant Extracts, Chinese[Title/Abstract]) OR (Chinese Plant Extracts[Title/Abstract]) OR (Extracts, Chinese Plant[Title/Abstract]) OR (Oriental Medicine, Traditional[Title/Abstract]) OR (Medicine, Traditional Oriental[Title/Abstract]) OR (Traditional Oriental Medicine[Title/Abstract]) OR (Traditional Oriental Medicines[Title/Abstract]) OR (Traditional Medicine, Oriental[Title/Abstract]) OR (Traditional East Asian Medicine[Title/Abstract]) OR (Medicine, Traditional, East Asia[Title/Abstract]) OR (Traditional Medicine, East Asia[Title/Abstract]) OR (Traditional Far Eastern Medicine[Title/Abstract]) OR (East Asian Traditional Medicine[Title/Abstract]) OR (Oriental Traditional Medicine[Title/Abstract]) OR (Medicine, Oriental Traditional[Title/Abstract]) OR (East Asian Medicine[Title/Abstract]) OR (East Asian Medicines[Title/Abstract]) OR (Medicine, East Asian[Title/Abstract]) OR (Oriental Medicine[Title/Abstract]) OR (Medicine, Far East[Title/Abstract]) OR (East Medicine, Far[Title/Abstract]) OR (East Medicines, Far[Title/Abstract]) OR (Far East Medicine[Title/Abstract]) OR (Far East Medicines[Title/Abstract]) OR (Medicines, Far East[Title/Abstract]) OR (Medicine, East Asia[Title/Abstract]) OR (Asia Medicines, East[Title/Abstract]) OR (East Asia Medicine[Title/Abstract]) OR (East Asia Medicines[Title/Abstract]) OR (Medicines, East Asia[Title/Abstract]) OR (Medicine, Oriental[Title/Abstract]) |
|  | 5. | 3 and 4 |
| Embase | 1. | 'Psoriasis'/exp |
|  | 2. | 'Psoriases' OR 'Pustulosis of Palms and Soles' OR 'Pustulosis Palmaris et Plantaris' OR 'Palmoplantaris' OR 'Pustulosis' OR 'Pustular Psoriasis of Palms and Soles' |
|  | 3. | 1 or 2 |
|  | 4. | 'Chinese medicine'/exp |
|  | 5. | 'Chinese medicinal formula'/exp |
|  | 6. | 'Chinese drug'/exp |
|  | 7. | 'Traditional medicine'/exp |
|  | 8. | 'Traditional Chinese Medicine' OR 'Chung I Hsueh' OR 'Hsueh, Chung I' OR 'Traditional Medicine, Chinese' OR 'Zhong Yi Xue' OR 'Chinese Traditional 'Medicine' OR 'Chinese Medicine, Traditional' OR 'Chinese Drugs, Plant' OR 'Chinese Herbal Drugs' OR 'Herbal Drugs, Chinese' OR 'Plant Extracts, Chinese' OR 'Chinese Plant Extracts' OR 'Extracts, Chinese Plant' |
|  | 9. | 4 or 5 or 6 or 7 or 8 |
|  | 10. | 3 and 9 |
| Cochrane | 1. | MeSH descriptor: [Psoriasis] explode all trees |
|  | 2. | MeSH descriptor: [Medicine, Chinese Traditional] explode all trees |
|  | 3. | (Psoriases):ti,ab,kw |
|  | 4. | (Pustulosis of Palms and Soles):ti,ab,kw |
|  | 5. | (Pustulosis Palmaris et Plantaris):ti,ab,kw |
|  | 6. | (Palmoplantaris Pustulosis):ti,ab,kw |
|  | 7. | (Pustular Psoriasis of Palms and Soles):ti,ab,kw |
|  | 8. | #3 OR #4 OR #5 OR #6 OR #7 |
|  | 9. | #8 OR #1 |
|  | 10. | (Traditional Chinese Medicine):ti,ab,kw |
|  | 11. | (Chung I Hsueh):ti,ab,kw |
|  | 12. | (Hsueh, Chung I):ti,ab,kw |
|  | 13. | (Traditional Medicine, Chinese):ti,ab,kw |
|  | 14. | (Zhong Yi Xue):ti,ab,kw |
|  | 15. | (Chinese Traditional Medicine):ti,ab,kw |
|  | 16. | (Chinese Medicine, Traditional):ti,ab,kw |
|  | 17. | (Chinese drugs):ti,ab,kw |
|  | 18. | (Chinese herbal medicine):ti,ab,kw |
|  | 19. | (Chinese herbal drug):ti,ab,kw |
|  | 20. | #10 OR #11 OR #12 OR #13 OR #14 OR #15 OR #16 OR #17 OR #18 OR #19 |
|  | 21. | #2 OR #20 |
|  | 22. | #9 AND #21 |
| CNKI& CQVIP& Wanfang& CBM | 1. | '银屑病'or'牛皮癣'or'白疕'or'寻常型银屑病'or'点滴型银屑病'or'银屑病关节炎'or'脓疱型银屑病'or‘关节病型银屑病’ |
|  | 2. | '中草药'or'中医'or'中医药'or'中药'or'方剂 |
|  | 3. | '临床' |
|  | 4. | 1 and 2 and 3 |

**Table S3: Frequency of top 8 herbs used in the trails**

| Name of Herbs (Latin name) | Frequency | Total Frequency (%) | Cumulative Percentiles (%) |
| --- | --- | --- | --- |
| Rhizoma Smilacis Glabrae (Rhizome of *Smilax glabra* Roxb) | 9 | 7.03% | 7.03% |
| Radix Paeoniae Rubra (Root of *Paeonia veitchii* Lynch) | 7 | 5.47% | 12.50% |
| Radix Salviae Miltiorrhizae (Root and rhizome of *Salvia miltiorrhiza* Bunge) | 7 | 5.47% | 17.97% |
| Rhizoma Curcumae Aeruginosae  (Rhizome of *Curcuma zedoaria* (Christm.) Roscoe) | 6 | 4.69% | 22.66% |
| Radix Rehmanniae  (Root of *Rehmannia glutinosa* (Gaertn.) DC.) | 6 | 4.69% | 27.34% |
| Radix Arnebiae (Root of *Lithospermum erythrorhizon* Sieb. et Zucc.) | 6 | 3.91% | 31.25% |
| Caulis Spatholobi (Dried lianoid stem of *Spatholobus suberectus* Dunn) | 5 | 3.91% | 35.16% |
| Chinese angelica  (Root of *Angelica sinensis* (Oliv.) Diels.) | 5 | 3.91% | 39.06% |
